# Profile distribution of soil moisture response to precipitation on the Pisha sandstone hillslopes of China

**DOI:** 10.1038/s41598-020-65829-w

**Published:** 2020-06-04

**Authors:** Pan Zhang, Peiqing Xiao, Wenyi Yao, Guobin Liu, Weiying Sun

**Affiliations:** 1 Key Laboratory of Soil and Water Loss Process and Control on the Loess Plateau of Ministry of Water Resources, Yellow River Institute of Hydraulic Research, Zhengzhou, 450003 China; 2 Research Center of Soil and Water Conservation and Ecological Environment, Chinese Academy of Sciences and Ministry of Education, Yangling, 712100 China

**Keywords:** Environmental impact, Hydrology

## Abstract

The Pisha sandstone area in China is located on the upper and middle reaches of the Yellow River, which is a region with some of the most severe soil erosion in both the Loess Plateau and in the world. Soil moisture is an important link between rainfall, surface water, and groundwater, and it plays a critical role in vegetative growth, ecosystem health, and the restoration of degraded vegetation. This study investigated the dynamic characteristics of soil moisture and its influencing factors in the hillslopes of the Pisha sandstone area using mathematical statistics and hydrochemical analysis methods. The results resolved that precipitation is the major direct source of soil moisture. Soil moisture fluctuated with precipitation, but the response time of these fluctuations was directly related to the antecedent soil moisture. Thus, while precipitation events increase the soil moisture content of the Pisha sandstone, they will not change the vertical distribution of moisture in the soil profile. The positive effect of precipitation on soil moisture was obvious in the soil layers above 50 cm, but deep soil moisture was less responsive to precipitation.

## Introduction

Soil moisture is an important link between rainfall, surface water, and groundwater, with spatio-temporal variations due to the influence of a range of hydrological processes^1,2^, including infiltration, runoff, wind erosion, freeze-thaw erosion, and sediment and pollutant transport. Especially in arid and water-scarce areas, soil moisture plays an important role in vegetative growth, the evolution of ecological patterns and controls, and the restoration of degraded vegetation^3^. The response of soil moisture to precipitation is an important component of the hydrological cycle. This is especially true in the context of global climate change, which manifests in increasingly unpredictable rainfall events. The response of soil moisture to precipitation in arid areas has thus attracted more and more attention from researchers worldwide. Therefore, understanding the relationship between soil moisture and precipitation is crucial to the efficient use of our limited water resources^4^.

In order to understand soil moisture dynamics, it is necessary to study the process of rainfall infiltration, runoff generation, and soil water redistribution. Previous studies covering different climatic zones have yielded a range of results. For example, Shen *et al*.^5^ studied the Tibetan Lhasa valley in the semi-arid monsoon climate zone of the Qinghai-Tibet Plateau and showed that the response of soil moisture to precipitation was different in different soil layers. The response of the 0–5 cm soil layer was rapid and direct, the 5–20 cm soil layer responses were delayed, and the response in the 20–40 cm soil layers was not obvious. Yang *et al*.^6^ explored how precipitation affects soil water at various soil depths of karst hillslopes and showed that the effects of precipitation on soil moisture could last two (0–50 cm) to five (70–100 cm) weeks. Ma *et al*.^7^ studied the response of soil moisture to fluctuations of precipitation on different time scales in grassland, shrub, and farmland ecosystems in the arid Qinghai Lake basin area. They found that the response of soil moisture to precipitation was about one month earlier in shallow layers (10 cm, 30 cm) than in deep layers (60 cm, 90 cm). He *et al*.^8^ quantitatively analyzed the response characteristics of soil moisture by layer to different intensity rainfall events in grassland and meadow systems in the Qilian Mountains. Their results showed that although total precipitation, frequency of rainfall events, and vegetation types were different, the response characteristics of the soil moisture to rainfall in the grassland and meadow layers were similar. Wang *et al*.^9^ used a watershed in the Loess Plateau to study the response characteristics of soil moisture to precipitation and analyzed the effects of different vegetation covers on rainfall infiltration. They found that different land cover types significantly affected the infiltration of precipitation. Liu *et al*.^10^ studied the characteristics of precipitation and the response of soil moisture fluctuations in the desert area of the Heihe River basin and found there were obvious differences in soil moisture characteristics before and after precipitation events, but the differences gradually decreased with the deepening of soil layer. Despite the above efforts to characterize spatial-temporal variations in soil moisture related to rainfall, most studies were based on laboratory experiments of artificial rainfall. The small number of field studies currently lack data on dynamic hydrological processes, probably due to the difficulty of long-term dynamic monitoring.

The Pisha sandstone area is located in wind-water-freeze-thaw erosion crisscross region on the Loess Plateau of China, where the freezing-thawing effect plays an important role in severe erosion. Soil moisture is a key factor that affects freezing-thawing erosion, so it is very important for the study of the erosion mechanism in this area. The response of soil moisture to precipitation is closely related to soil texture and rainfall characteristics^11^. In semi-arid rocky mountainous areas like Ordos, the physical and hydrological properties of the soil are deeply affected by the parent bedrock because of its shallow soil layers. The highly-stratified rock strata are very heterogeneous in this area, thus the soil moisture response to rainfall is more complicated. Freezing-thawing erosion mainly affects the shallow soil layer within 50 cm from the surface, and the soil moisture here is especially variable under the influence of weathering factors such as rainfall and temperature. But up to now, there is relatively little continuous and automatic monitoring of soil moisture in this area, the understanding of dynamic changes in the soil water profile, rainfall response processes, the physics of soil water distribution and migration is insufficient. In order to correct this lack of information and understanding, we selected the special geomorphic unit of the typical hillslope in the Pisha sandstone area and used high-resolution, continuous monitoring of the soil moisture profile to capture any dynamic changes of soil moisture and its influencing factors under typical rainfall conditions. The present study has three main objectives: 1) to systematically acquire information about the spatio-temporal variability and temporal stability characteristics of soil profile moisture at the hillslope scale; 2) to gain a profound understanding of the soil moisture distribution in different soil layers in response to different precipitation events in the Pisha sandstone area; and 3) to evaluate the correlation between precipitation events and soil moisture.

## Materials and methods

### Study site and its soil properties

**Figure 1:**
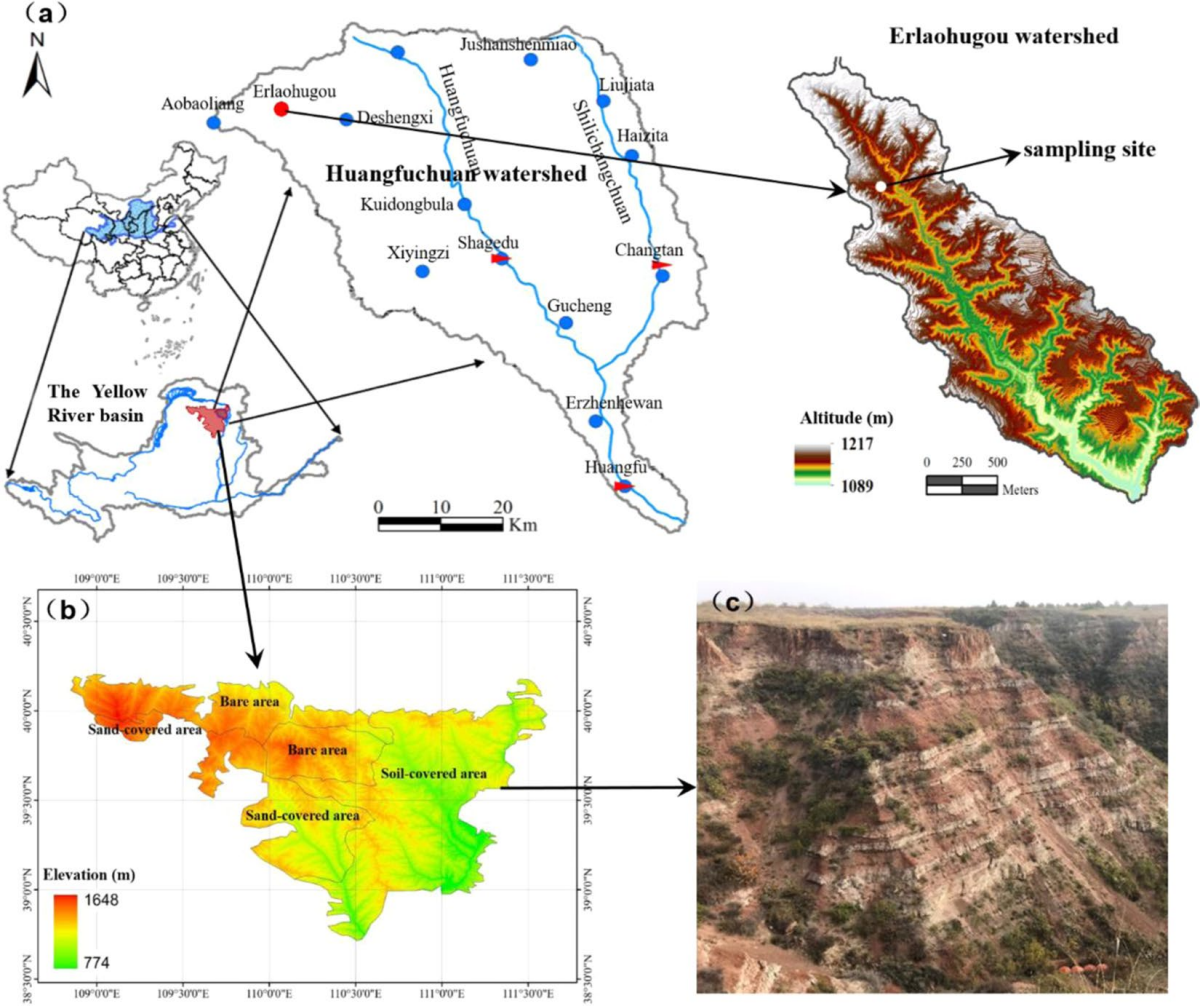
Location of the study area, (**a**,**b**) were generated by importing vector files in shp format in ArcGIS 10.7.

Figure 1. shows the location of the study area. The Pisha sandstone area is approximately 16,700 km^2^, and is widely distributed in the semi-arid areas of the Shanxi and Shaanxi Provinces, and in the Yellow River Basin in Inner Mongolia^12,13^. According to ground materials, the area can be divided into three types: soil-covered (8,400 km^2^), sand-covered (3,800 km^2^), and bare areas (4,500 km^2^) (Fig. 1b). Pisha sandstone is an interbedded rock belonging to the continental clastic sedimentary rock class, consisting of sandstone, sand shale, and mudstone gradually formed in the Paleozoic Permian, Mesozoic Triassic, Jurassic, and Cretaceous periods (Fig. 1c)^14,15^. As the rock layer covered by the Pisha sandstone is small in thickness and low in pressure, it has weak diagenesis and low structural strength. The Pisha sandstone is hard like rock when it is dry, but can collapse into sand within a minute when immersed in water^16^. Due to the special physical properties of the Pisha sandstone, coupled with the hydraulic, wind, and freeze-thaw erosion effects of the Pisha sandstone area, this area has severe soil erosion and an extremely harsh environment^17–20^. Multiple types of erosion occur alternately in this area, resulting in compound erosion; the erosion modulus in this area is as high as 30,000–40,000 t/(km^2^·a). The Pisha sandstone is broken into sediment under the action of compound erosion and these eroded sediments are deposited in the Yellow River, raising the altitude of the riverbed^21,22^. Consequently, the Pisha sandstone area presents a deserted landscape with sparse vegetation and numerous gullies that has been cited as having the most severe soil erosion in both the Loess Plateau and in the world^20,23,24^.

The study site was a hillslope (mean gradient of 37°) located in the Erlaohugou watershed (3.23 km^2^) in zhun-ge-er Banner, Inner Mongolia Ordos, China (39°47′39′′ N, 110°36′3′′E; Fig. 1a). The hillslope is within the soil-covered Pisha sandstone area. The topography in this area belongs to the transitional zone of the Loess plateau and the Ordos plateau; the surface covering was loess soil, and the sandstone was exposed in both the valley and the slope, with the exposed area covering over 70% of the area. The hillslope was approximately 92 m long and 32 m wide. The Pisha sandstone was widely exposed on this slope, and the vegetation coverage was only 1.7%.

The study area has an arid and semi-arid temperate continental monsoon climate with cold winters, hot summers, and more wind and sand in the winter and spring^25^. The area has an average annual temperature of 6.2 °C, a wind speed of 3.6 m/s, 2900 h of sunlight^26^, a relative humidity of 52%, mean annual precipitation of 386 mm/year, and mean annual potential evaporation of 2234 mm/year (about 5.8-times the amount of precipitation). Soil types in this area include dark loess soil, loess soil, chestnut soil, and aeolian soil. The loess soil at the study site was mainly distributed on the top of the hillslope, and had a silt loam texture in the upper 1~2 m layer with a mean soil bulk density of 1.3 g/cm^3^; the stable infiltration was 66 mm/h. The hillslope was bare Pisha sandstone with a soil bulk density of 1.36–1.58 g/cm^3^, and a stable infiltration of 47 mm/h. The particle size distribution of the loess soil and the Pisha sandstone soil is shown in Table 1. The particle size of the Pisha sandstone soil was coarser than the loess soil, especially the content of coarse sediment with particle sizes >0.05 mm, which is much higher than that of the loess; the organic matter content of the Pisha sandstone was lower than that of the loess.

**Table 1:** Soil characteristics based on sample analysis.

Soil type	Particle size (mm)	Loess	Pisha sandstone
Soil composition (%)	0.005~0.01	43.40	2.96
	0.01~0.05	35.45	15.03
	>0.05	21.15	78.58
Texture		Silt loam	cross-bedded sandstone
Organic matter content (%)		0.32	0.106

### Soil moisture and precipitation monitoring

We selected two points (P1 and P2) on the hillslope for this study that had a similar vegetation cover (3%) and slope aspect. The two points were located in the middle and upper part of the slope, about 6 meters away from the top of the slope, and 7.5 meters apart from each other. The distribution of soil particles at these points was mainly sand, with consisted of 3.95% fine silt (0.005–0.01 mm), 13.8% coarse silt (0.01–0.05 mm), and 75.8% coarse sand (>0.05 mm). To monitor soil moisture at different depths, soil moisture sensors (ECH_2_O-5, Decagon Devices Inc., United States; precision: ±3%) were inserted into the soil profile at depths of 10, 20, 30, 40, and 50 cm. We then backfilled the soil profile with excavated soil, making sure to pack the soil to the approximate density of the surrounding soil. Soil moisture was measured hourly from March 1, 2018 until November 30, 2018. The ECH_2_O-5 sensors determine volumetric water content (VWC) by measuring the dielectric constant of the media using capacitance/frequency domain technology. Its 70 MHz frequency minimizes salinity and textural effects, making this sensor accurate in almost any soil or soil-less media, and can measure soil moisture without disturbing the soil structure. The measurement interval was an hour. Simultaneously, we measured the amount of precipitation in the study area by the dump bucket-type rain bucket produced by Onset Computer Corporation, USA. The measurement precision was 0.2 mm and the recording interval was 15 minutes.

### Data analysis

We calculated basic statistics for the soil moisture readings at each soil depth, including the mean, standard deviation (SD), coefficient of variation (CV), and maximum (Max) and minimum (Min) values. Among these, SD and CV are indicators that characterize the change range of soil moisture, soil profile was divided into four layers according to them: rapidly change layer (CV ≥ 30%, SD ≥ 4%), active layer (20% <CV < 30%, 3% <SD < 4), sub-active layer (10% <CV < 20%, 2% <SD < 3%), and relatively stable layer (CV ≤ 10%, SD ≤ 2%)^27^. Since it is difficult to satisfy both of these indicators at the same time, the soil moisture distribution along the profile is mainly divided according to CV^28^. Seasonal soil water storage variation was divided into three periods: consuming, supplying, and relatively stable periods^27,29^.

### Gray relational analysis (GRA)

Gray relational analysis (GRA) is a method of gray prediction, which can deal with limited and surface irregular data to find the characteristics of the system itself. Variations of soil moisture were affected by various factors such as precipitation, soil properties, and antecedent soil moisture. The various factors are not independent of each other, but are connected with each other to often have a synergistic effect on overall soil moisture dynamics. The degree of gray correlation for the soil moisture was the result of the combined effects of various factors affecting soil moisture, and can reflect the comprehensive effect of various factors affecting soil moisture. Therefore, for the first time, the GRA method was used to study the relationship between soil moisture and precipitation in different soil layers^30,31^. The procedures that follow were adopted.

Step 1: Dimensionless processing of the original data. Since the data collected in the experiment were often not uniform, in order to increase the comparability of the data, it was necessary to deal with the original data without dimensions. In this paper, the maximum range transformation method was used. See Eq. (1):

1 $${x}_{i}^{\ast }(k)=\frac{\max ({x}_{i}^{0}(k))-{x}_{i}^{0}(k)}{\max ({x}_{i}^{0}(k))-\,{\rm{\min }}({x}_{i}^{0}(k))},$$

where ${x}_{i}^{0}(k)$ is the actual sequence; ${x}_{i}^{\ast }(k)$ is the sequence after data pre-processing; ${x}_{i}^{0}(k=1)$ is the first value of the sequence; ${\rm{\max }}({x}_{i}^{0}(k))$ is the largest value of ${x}_{i}^{0}(k)$; and ${\rm{\min }}({x}_{i}^{0}(k))$ is the smallest value of ${x}_{i}^{0}(k)$.

Step 2: Calculate the degree of the gray relation coefficient (*ξ*(*k*)) of the comparison sequence (${x}_{i}^{0}(k)$) and the reference sequence (${x}_{i}^{\ast }(k)$), which shows the relationship between the desired and actual normalized experimental results. See Eq. (2):

2 $$\xi (k)=\frac{{\Delta }_{{\rm{\min }}}+\xi {\Delta }_{{\rm{\max }}}}{\Delta {0}_{i}(k)+\xi {\Delta }_{{\rm{\max }}}},$$

where $\Delta {0}_{i}(k)$ is the absolute variance between ${x}_{i}^{0}(k)$ and ${x}_{i}^{\ast }(k)$, which is also known as the deviation sequence; ${\Delta }_{{\rm{\min }}}$ is the minimum of $\Delta {0}_{i}(k)$; ${\Delta }_{{\rm{\max }}}$ is the maximum of $\Delta {0}_{i}(k)$; $\xi$ is the differentiating coefficient; and $\,\xi \in [0,\,1]$ and $\,\xi$ = 0.5 are the widely accepted values.

Step 3: The number of correlation coefficients was the same as the number of reference sequences. In order to improve the reliability of the analysis results, the number of selected reference sequences will be greater, so the number of correlation coefficients will also be greater, resulting in more scattered information. In order to facilitate analysis and comparison, the correlation coefficient at each time was often converted into a quantity value by means of the average value. See Eq. (3):

3 $${\gamma }_{i}=\frac{1}{n}\mathop{\sum }\limits_{k=1}^{n}{w}_{k}{\xi }_{i}(k),$$

where ${w}_{k}$ denotes the normalized weightage of factor *k*; and *n* is the number of response variables.

## Results and discussion

### Spatio-temporal distribution of soil moisture on profiles

The mean, standard deviation (SD), coefficients of variation (CV), and extreme maximum and minimum values of the moisture contents of the soil profiles were used to represent the spatio-temporal variability in soil moisture (Table 2). Season and spatial location obviously affected soil moisture.

**Table 2:** Temporal statistics for the spatial mean soil moisture, the corresponding standard deviations (SD) and the coefficients of variation (CV) for the various soil layers (March 2018 –November 2018).

Site	Soil depth (cm)	Wet season					Dry season				
		Max (%)	Min (%)	Mean (%)	SD (%)	CV (%)	Max (%)	Min (%)	Mean (%)	SD (%)	CV (%)
P1	0–10	66.8	25.1	27.3	4.2	15.3	26.9	19.8	23.4	3.3	14.2
	10–20	65.5	35.8	41.2	3.8	9.3	44.6	28.9	36.1	3.5	9.7
	20–30	53.4	29.7	32.5	2.5	7.8	33.7	25.6	29.6	1.8	6.2
	30–40	50.7	27.1	33.1	2.5	7.5	38.0	22.6	28.5	1.9	6.6
	40–50	53.2	22.7	31.0	3.1	9.9	44.8	21.4	27.2	1.8	6.5
P2	0–10	27.8	16.4	20.6	2.7	13.3	21.6	14.1	18.0	1.8	9.9
	10–20	53.3	33.5	39.6	4.6	11.5	39.4	24.4	32.8	2.1	6.3
	20–30	46.7	32.8	38.0	3.3	8.6	35.5	24.1	31.5	1.8	5.8
	30–40	41.9	32.4	35.6	2.3	6.4	33.1	25.9	31.2	1.1	3.4
	40–50	43.7	28.9	31.1	1.5	4.7	30.4	22.3	28.1	1.5	5.2

During wet periods, the mean soil moisture in the P1 site profiles showed a trend of first increasing and then decreasing with depth, with values of 27.3%, 41.2%, 32.5%, 33.1%, and 31.0% for the 0–10, 10–20, 20–30, 30–40, and 40–50 cm depths, respectively. The same trend was shown at the P2 site with values of 20.6%, 39.6%, 38.0%, 35.6%, and 31.1% in the 0–10, 10–20, 20–30, 30–40, and 40–50 cm depths, respectively. Soil moisture in the wet season (from June to September), was significantly higher than that in dry season (from October to May of the next year) at both sites. For example, the mean soil moisture values at the P1 and P2 sites in the wet season were 13.9% and 16.5% higher, respectively, than those in dry season. The statistical data showed that soil moisture varied significantly in the different seasons, but not at the different spatial points, which indicated that seasonal variations have a strong influence on soil moisture, while spatial variation has a weak influence on it.

**Figure 2:**
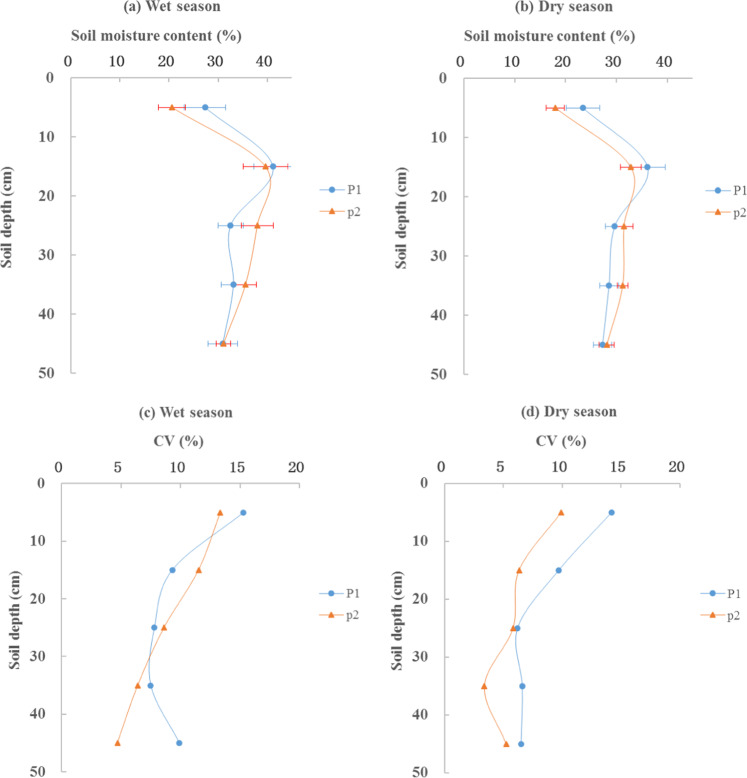
Mean values of soil moisture with error bars showing standard deviation (**a,b**) and their corresponding coefficient of variation (CVs) (**c,d**) from 0 to 50 cm depths in the soil profile of each site separated into wet and dry periods.

Figure 2 shows mean soil moisture and corresponding values of CV over time in the 0–50 cm soil profiles of the hillslope. Whether in the dry or wet season, soil moisture in the 0–17 cm soil layer increased with soil depth, and achieved its maximum at both the P1 and P2 sites at a depth of approximately 17 cm (Fig. 2a,b). We then used the CV to describe the degree to which soil moisture changed with time (Fig. 2c,d). High soil moisture CV values were mainly detected in the shallow layers (0–20 cm) of the two sites, and these values decreased with increased soil depth. In the 20–50 cm layer, the values of SD and CV were lower than those in the other layers, and remained stable. The spatial variability of the SD and CV values of the mean soil moisture content changed more in the shallow soil layers compared with the deeper layers; this indicated that the temporal changes in the spatial mean soil moisture were mainly present in the shallow soil layers (0–20 cm). This result corresponded to the findings of Li *et al*.^32^, Penna *et al*.^33^ and Gao *et al*.^34^, who all found that shallow layers exhibit higher soil moisture variability over time than do deep layers. The reason may be due to the fact that precipitation, evaporation, and infiltration have more significant effects on shallow soil layers.

### Dynamic response of soil moisture to precipitation

Table 3 shows the maximum and minimum soil moisture at different soil depths. Variance analysis showed that soil water infiltration was affected by antecedent soil moisture, and there were significant differences between the minimum soil moisture and maximum soil moisture at different depths. For the eight precipitation events that occurred in the study area from March 1 to November 30, 2018, soil moisture was the lowest in March and the highest in July.

**Table 3:** Minimum and maximum soil moisture at different soil depths.

Site	Soil depth (cm)	Date of minimum soil moisture	Minimum soil moisture (%)	Date of maximum soil moisture	Maximum soil moisture (%)
P1	0–10	Mar. 25	19.8	Jul. 16	66.8
	10–20	Mar. 25	28.9	Jul. 16	65.5
	20–30	Mar. 25	25.6	Jul. 16	53.4
	30–40	Mar. 25	22.6	Jul. 16	50.7
	40–50	Oct. 12	21.4	Jul. 16	53.2
P2	0–10	Mar. 25	14.1	Jul. 15	27.8
	10–20	Mar. 25	24.4	Jul. 15	53.3
	20–30	Mar. 25	24.1	Jul. 16	46.7
	30–40	Mar. 25	25.9	Jul. 16	41.9
	40–50	Mar. 25	22.3	Jul. 16	43.7

**Figure 3:**
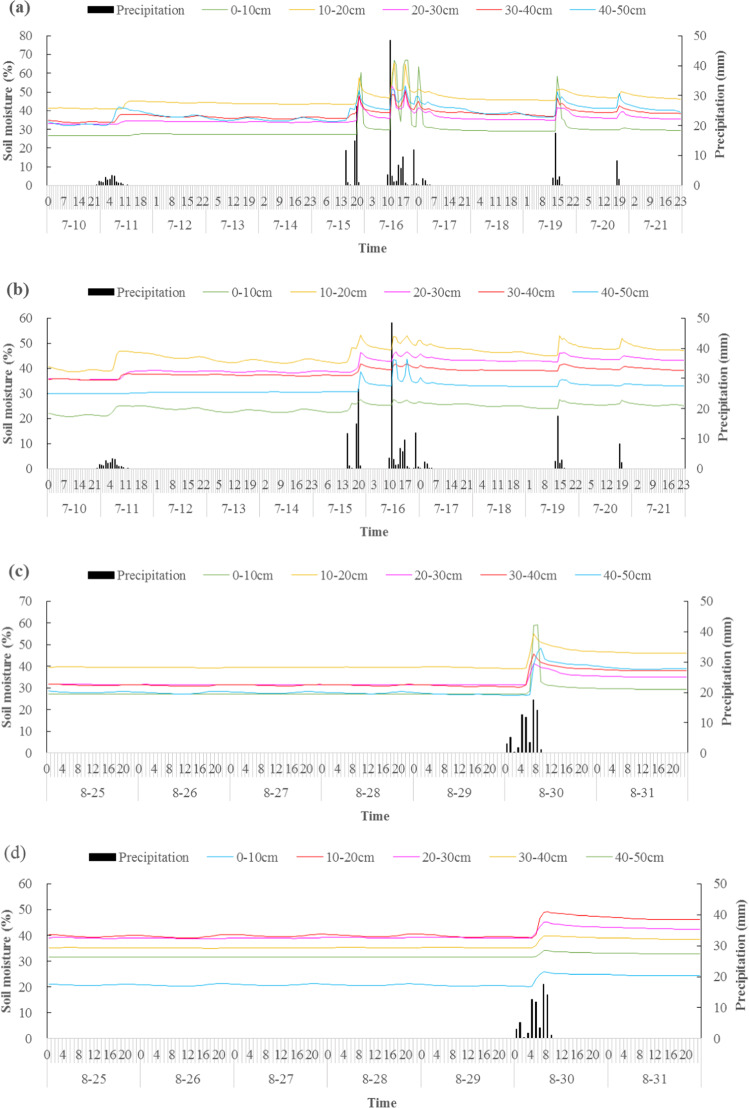
Temporal variation in soil water contents of the two sites, P1 (**a**,**c**) and P2 (**b**,**d**), from March 1, 2018 - November 30, 2018.

Figure 3 shows the dynamic changes of soil moisture with time at different depths throughout the study. For the measurements in the 0–50 cm depths, soil moisture was highly variable and closely related to the precipitation events. Due to the coarse sediment and high permeability of the Pisha sandstone, the occurrence time of the soil moisture peak was closely related to the occurrence time of the precipitation peak, which indicated that the time required to infiltrate the top 50 cm of soil was very short.

In July, there were five precipitation events for a total precipitation amount of 210.2 mm. The heaviest precipitation in a single precipitation event occurred on July 16 with 93.8 mm, as shown in Fig. 3. When precipitation reached a certain soil layer, the soil moisture sensor responded; we observed five responses from within the 0–50 cm soil depth in the P1 and P2 sites, and for each event, the response time of each soil layer was almost the same. This suggests that all five precipitation events were intense enough to reach even the 50-cm soil depth, and that the soil infiltration rate was very fast. Moreover, in the first two precipitation events, the soil moisture response time was about 4–6 h after the precipitation events. In the later three precipitation events, the response time was greatly reduced, and the change in soil moisture was usually less than 2 h behind the precipitation events. This was due to the long-term drought before the first two precipitation events, which led to serious deficiencies in soil moisture. This pattern suggests that the soil moisture response time to precipitation was directly related to the antecedent soil moisture; thus, the first two precipitation events increased the antecedent soil moisture, and the increased antecedent soil moisture significantly shortened the response time during later rainfall events.

In August, there was only one precipitation event, with a total precipitation amount of 70.8 mm. We observed that the soil moisture throughout the entire 50-cm soil layer was closely related to the precipitation event, and the response time of each soil layer was almost the same, about 5 h after the precipitation event. The reason for the increased response time was that there was no rainfall in the area from July 20 to August 30, so the lower antecedent soil moisture increased the response time during the August rainfall event. Precipitation events and their responses at depths less than 50 cm indicated that rainfall was the dominant factor affecting soil moisture in the Pisha sandstone area, and antecedent soil moisture had a significant impact on response time. These results are in agreement with the findings of Li *et al*.^32^, Penna *et al*.^33^ and Gao *et al*.^34^.

### Soil moisture distribution during the wet season

**Figure 4:**
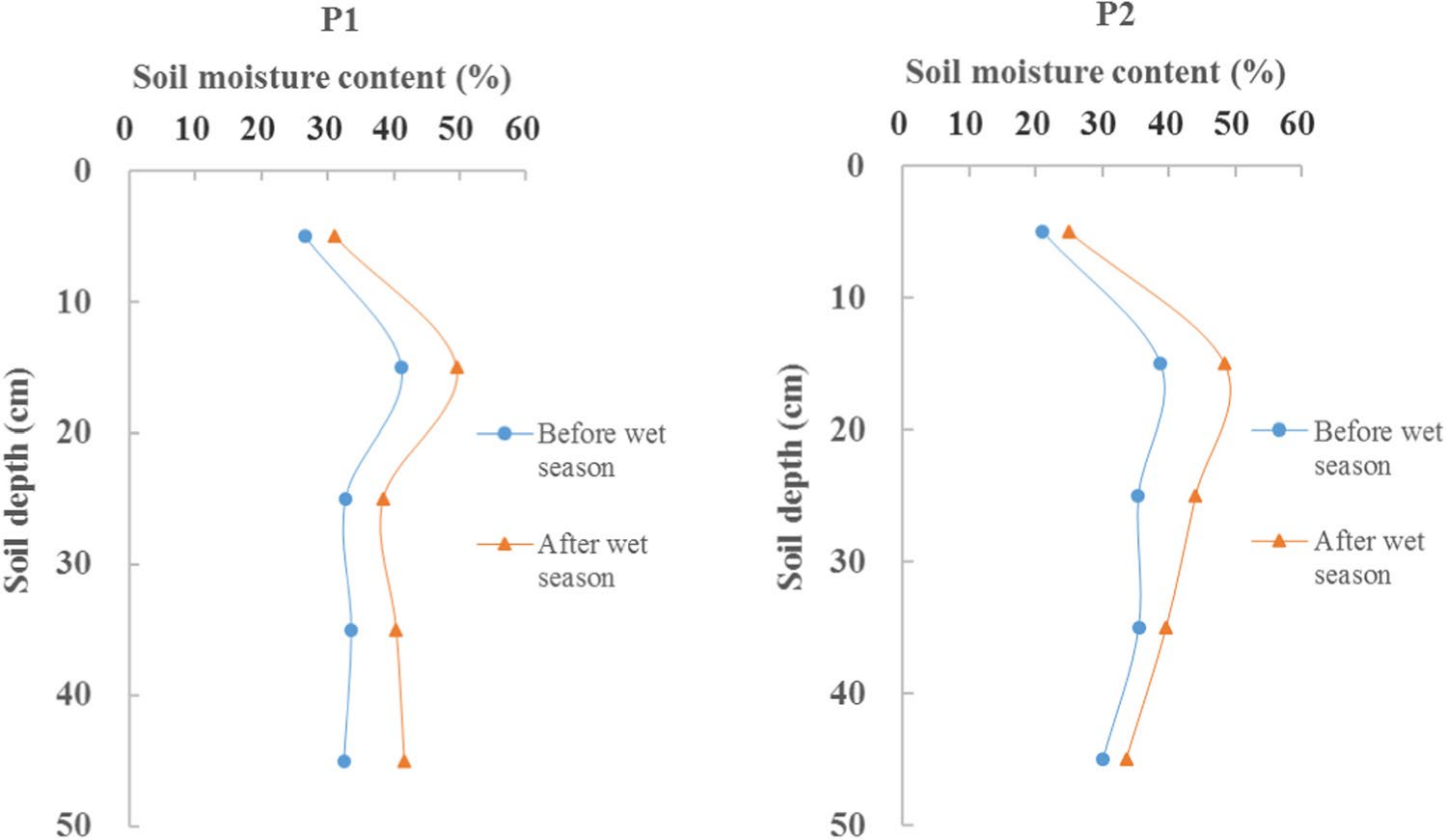
Vertical variations in mean profile soil moisture around the wet season in the P1 and P2 sites.

Figure 4 shows the characteristics of soil moisture variation before and after the wet season. During the observation period, 281 mm of the total precipitation fell during the wet season. From Jun. 1 to Aug. 30, 2018, there were six precipitation events. The vertical distribution of soil moisture in the five top soil layers was basically the same in the time periods surrounding the wet season. However, the mean soil moisture for the P1 and P2 sites increased by 7% and 6%, respectively after the wet season compared with that before wet season. In this study, soil moisture fluctuated with precipitation, but only small differences were found between the wet and dry seasons. This suggests that precipitation events increased the soil moisture content of the Pisha sandstone, but it did not change the vertical distribution of soil moisture in the soil profile. This may be related to the soil texture of the Pisha sandstone.

### GRA of precipitation and soil moisture

We divided the soil profile into five levels of *L*_1_ (0–10 cm), *L*_2_ (10–20 cm), *L*_3_ (20–30 cm), *L*_4_ (30–40 cm), and *L*_5_ (40–50 cm). The soil moistures of each layer from March to November were set to ${{\rm{X}}}_{1}=\{{{\rm{X}}}_{1}({\rm{k}})|{\rm{k}}=3,4,\cdots 11\}$, ${{\rm{X}}}_{2}=\{{{\rm{X}}}_{2}({\rm{k}})|{\rm{k}}=3,4,\cdots 11\}$, ${{\rm{X}}}_{3}=\{{{\rm{X}}}_{3}({\rm{k}})|{\rm{k}}=3,4,\cdots 11\}$, ${{\rm{X}}}_{4}=\{{{\rm{X}}}_{4}({\rm{k}})|{\rm{k}}=3,4,\cdots 11\}$, and ${{\rm{X}}}_{5}=\{{{\rm{X}}}_{5}({\rm{k}})|{\rm{k}}=3,4,\cdots 11\}$. First, the surface layer was taken as the reference sequence, and the other layers as the comparison sequence, then we calculated the gray correlation coefficient between them (recorded as *R*_12_, *R*_13_, *R*_14_, and *R*_15_, respectively). Then, the second layer was taken as the reference sequence, and the other layers as the comparison sequence in order to calculate the gray correlation coefficient between them (recorded as *R*_21_, *R*_23_, *R*_24_, and *R*_25_, respectively), and so on. The GRA method was used to calculate the correlation degree and ranking of these parameters. When calculating the gray correlation coefficient between a parameter and other parameters, we took one parameter as the reference sequence and other parameters as the analysis sequence.

Table 4 shows the degrees of gray correlation and the order of the 10 combinations between the five soil layers. At the P1 site, the soil layers with high gray correlation degrees were *R*_23_ > *R*_12_ > *R*_13_, and the soil layers with lower gray correlations were *R*_35_ < *R*_45_ < *R*_15_. At the P2 site, the soil layers with high gray correlation degrees were *R*_12_ > *R*_13_ > *R*_23_, and the soil layer with lower gray correlations were *R*_34_ < *R*_24_ < *R*_45_. This showed that there was a significant correlation between the soil moisture in each layer, but there were also differences. The correlation between the surface soil moisture and the subsurface soil moisture was relatively close, followed by the middle soil moisture. However, the gray correlation of the deep soil moisture was weak, indicating that the middle soil moisture mainly came from the seepage of the surface layer, and as the surface seepage caused by precipitation can reach the middle layer (30 cm) directly, then most of that moisture can also reach the deeper layers (below 30 cm).

**Table 4:** Gray correlation degrees (*R*) between the soil moisture in different soil layers.

*R* of P_1_						*R* of P_2_				
	*L*_1_	*L*_2_	*L*_3_	*L*_4_	*L*_5_	*L*_1_	*L*_2_	*L*_3_	*L*_4_	*L*_5_
*L*_1_	1.000	0.907	0.879	0.805	0.624	1.000	0.920	0.882	0.844	0.846
*L*_2_		1.000	0.916	0.852	0.871		1.000	0.849	0.685	0.784
*L*_3_			1.000	0.638	0.534			1.000	0.573	0.778
*L*_4_				1.000	0.538				1.000	0.744
*L*_5_					1.000					1.000

Using the monthly precipitation data as a reference sequence (which was ${{\rm{X}}}_{0}=\{{{\rm{X}}}_{0}({\rm{k}})|{\rm{k}}=3,4,\cdots 11\}$) and the monthly soil moisture data in the different soil layers as the comparison sequences (which were ${{\rm{X}}}_{1,2,3,4,5}=\{{{\rm{X}}}_{1,2,3,4,5}({\rm{k}})|{\rm{k}}=3,4,\cdots 11\}$), we calculated the gray correlation coefficient between them (recorded as *R*_1_, *R*_2_, *R*_3_, *R*_4_, and *R*_5_ respectively). Table 5 shows the gray correlation degree between precipitation and soil moisture in the different soil layers.

**Table 5:** Gray correlation degree (*R*) between precipitation and soil moisture in the different soil layers.

	*R*_1_	*R*_2_	*R*_3_	*R*_4_	*R*_5_
P_1_	0.579	0.562	0.550	0.556	0.561
P_2_	0.598	0.582	0.576	0.564	0.572

From Table 5 we can see that the effect of precipitation on soil moisture was most obvious in the soil layers above 50 cm. However, the soil layers responded slightly differently to precipitation. At the P1 site, the order of the gray correlation degree with precipitation was as follows: *R*_1_ > *R*_2_ > *R*_5_ > *R*_4_ > *R*_3_. At the P2 site, the order of the gray correlation degree with precipitation was as follows: *R*_1_ > *R*_2_ > *R*_3_ > *R*_5_ > *R*_4_. In general, deep soil moisture was less responsive to precipitation than shallower soils.

## Conclusions

We analyzed high-resolution (hourly) variations of soil moisture in multiple soil layers in two slope positions (P1 and P2) from March 1, 2018 to November 30, 2018. The results indicated that mean soil moisture was higher in the wet season than in the dry season. Precipitation was the direct source of soil moisture, and the variation of soil moisture were the result of the combined actions of precipitation, infiltration, and soil evaporation. Soil moisture fluctuated with precipitation, but very few differences were found between precipitation events. The response time of soil moisture to precipitation was directly related to the antecedent soil moisture. The response time of soil moisture in the upper 50 cm was within 5 h after a precipitation event, and due to the high permeability of the Pisha sandstone, the response time of each soil layer was almost the same. Precipitation events increased the content of soil moisture of the Pisha sandstone, but it did not change the vertical distribution of moisture in the soil profile. There was a significant correlation between soil moisture in each layer, and correlations between surface soil moisture and subsurface soil moisture was relatively strong. The positive effect of precipitation on soil moisture was obvious in the soil layers above 50 cm, but deep soil moisture was less responsive to precipitation.

## References

[1] Huang X (2016). Soil moisture dynamics within soil profiles and associated environmental controls. Catena.

[2] Liu JT (2018). Study on the dynamic characteristics of groundwater in the valley plain of Lhasa City. Environ. Earth Sci..

[3] Rodríguez-Iturbe, I. & Porporato, A. Ecohydrology of Water-controlled Ecosystems: Soil Moisture and Plant Dynamics. *Cambridge University Press*, England (2005).

[4] Cheng YB, Zhan HB, Yang WB, Bao F (2018). Deep soil water recharge response to precipitation in Mu Us Sandy Land of China. Water Sci. Eng..

[5] Shen Z, Hua M, Lu J, Zhang C, Fang J (2016). Response of soil moisture to precipitation in the valley mountain Lhasa, Tibet. China Rural. Water Hydropower.

[6] Yang J, Chen HS, Nie YP, Wang KL (2019). Dynamic variations in profile soil water on karst hillslopes in Southwest China. Catena.

[7] Ma WM, Zhang XC (2016). Effect of Pisha sandstone on water infiltration of different soils on the Chinese Loess Plateau. J. Arid. Land..

[8] He Z, Zhao W, Liu H, Chang X (2012). The response of soil moisture to rainfall event size in subalpine grassland and meadows in a semi-arid mountain range: A case study in northwestern China’s Qilian Mountain. J. Hydrol..

[9] Wang S, Fu B, Gao G, Liu Y, Zhou J (2013). Responses of soil moisture in different land cover types to rainfall events in a re-vegetation catchment area of the Loess Plateau, China. Catena.

[10] Liu B, Zhao WZ, Chang XX, Li SB (2011). Response of soil moisture to rainfall pulse in desert region of the Heihe River basin. J. desert Res..

[11] Heathman GC, Cosh MH, Merwade V, Han E (2012). Multi-scale temporal stability analysis of surface and subsurface soil moisture within the Upper Cedar Creek Watershed, Indiana. Catena.

[12] Ma WM, Zhang XC (2016). Effect of Pisha sandstone on water infiltration of different soils on the Chinese Loess Plateau. J. Arid. Land..

[13] Ni HB, Zhang LP, Zhang DR, Wu XY, Fu XT (2008). Weathering of Pisha- sandstones in the wind-water erosion crisscross region on the Loess Plateau. J. Mt. Sci..

[14] Chen WD (2012). The Pisha sandstone in Yellow River basin- nonfarming land and watch as picture scroll. Natl Geogr. China.

[15] Martin MW, Jorge CR, Constantino MM (1999). Late Paleozoic to early Jurassic tectonic development of the high Andean principal cordillera, El Indio region, Chile (29-30°s). J. South. Am. Earth Sci..

[16] Bi CF, Wang FG, Li GF (2003). Experiment on sediment retention by plant “flexible dam” in gully in soft rock region. J. Sediment. Res..

[17] Cerda A (2017). Runoff initiation, soil detachment and connectivity are enhanced as a consequence of vineyards plantations. J. Environ. Manag..

[18] Guo J, Shi YC, Wu LJ (2015). Gravity erosion and lithology in Pisha sandstone in southern Inner Mongolia. J. Ground water Sci. Eng..

[19] Li CM, Zhang TT, Wang LJ (2014). Mechanical properties and microstructure of alkali activated Pisha sandstone geopolymer composites. Constr. Build. Mater..

[20] Wang, Y. C., Wu, Y. H. & Li, M. Study on water and soil loss and control methods in Pisha sandstone. Zhengzhou, China: *The Yellow River Water Conservancy Press*, China (in Chinese) (2007b).

[21] Yang FS (2014). Simulation of sediment retention effects of the double seabuckthorn plant flexible dams in the Pisha Sandstone area of China. Ecol. Eng..

[22] Zhang K, Xu MZ, Wang ZY (2009). Study on reforestation with seabuckthorn in the Pisha Sandstone area. J. Hydro-Environment Res..

[23] Wang, Y. C. *et al*. De- finition of arsenic rock zone borderline and its classification. *Science of Soil& Water Conservation* **5****(****1****)**, 14–18 (in Chinese) (2007a).

[24] Yang FS, Cao MM, Li HE, Wang XH, Bi CF (2014). Ecological restoration and soil improvement performance of the seabuckthorn flexible dam in the Pisha Sandstone area of Northwestern China. Solid. Earth Discuss..

[25] Liang Z (2018). A comprehensive method of erosion resistance and growth promotion for pisha sandstone. Int. J. Geomate.

[26] Xu YH, Yao XJ, Lu LB, Fan HJ (2015). The evaluation of ecoenvironmental sensitivity in Ordos City. Asian Agric. Res..

[27] Chen HS, Zhang W, Wang KL, Fu W (2010). Soil moisture dynamics under different land uses on karst hillslope in Northwest Guangxi, China. Environ. Earth Sci..

[28] Yu BW (2018). Soil moisture variations at different topographic domains and land use types in the semi-arid Loess Plateau, China. Catena.

[29] Zhang C (2013). Dynamics of soil profile water content in peak-cluster depression areas in karst region. China. J. Eco-Agric..

[30] Sun G, Guan X, Yi X (2018). Gray relational analysis between hesitant fuzzy sets with applications to pattern recognition. Expert. Syst. Appl..

[31] Zhang P, Yao WY, Liu GB, Xiao PQ (2019). Experimental study on soil erosion prediction model of loess slope based on rill morphology. Catena.

[32] Li TC, Shao MA, Jia YH, Jia XX, Huang LM (2018). Profile distribution of soil moisture in the gully on the northern Loess Plateau, China. Catena.

[33] Penna D, Brocca L, Borga M, Fontana DG (2013). Soil moisture temporal stability at different depths on two alpine hillslopes during wet and dry periods. J. Hydrol..

[34] Gao L, Shao MA (2012). Temporal stability of soil water storage in diverse soil layers. Catena.

